# The Road to Resistance in Forest Trees

**DOI:** 10.3389/fpls.2019.00273

**Published:** 2019-03-29

**Authors:** Sanushka Naidoo, Bernard Slippers, Jonathan M. Plett, Donovin Coles, Caryn N. Oates

**Affiliations:** ^1^ Division of Genetics, Department of Biochemistry, Genetics and Microbiology, Forestry and Agricultural Biotechnology Institute (FABI), University of Pretoria, Pretoria, South Africa; ^2^ Hawkesbury Institute for the Environment, Western Sydney University, Richmond, NSW, Australia

**Keywords:** genomics, breeding, candidate genes, endophytes, genetic engineering

## Abstract

In recent years, forests have been exposed to an unprecedented rise in pests and pathogens. This, coupled with the added challenge of climate change, renders forest plantation stock vulnerable to attack and severely limits productivity. Genotypes resistant to such biotic challenges are desired in plantation forestry to reduce losses. Conventional breeding has been a main avenue to obtain resistant genotypes. More recently, genetic engineering has become a viable approach to develop resistance against pests and pathogens in forest trees. Tree genomic resources have contributed to advancements in both these approaches. Genome-wide association studies and genomic selection in tree populations have accelerated breeding tools while integration of various levels of omics information facilitates the selection of candidate genes for genetic engineering. Furthermore, tree associations with non-pathogenic endophytic and subterranean microbes play a critical role in plant health and may be engineered in forest trees to improve resistance in the future. We look at recent studies in forest trees describing defense mechanisms using such approaches and propose the way forward to developing superior genotypes with enhanced resistance against biotic stress.

## Introduction

Forest trees in their native range are introduced and domesticated as plantation stock encounter various biotic and abiotic factors that influence their persistence and productivity. In recent years, introduced pests and pathogens have been increasing at an alarming rate (Santini et al., 2013; Wingfield et al., 2015; Hurley et al., 2016). In some cases, these invasions threaten tree species with local extinction, such as the fungal pathogen *Cryphonectria parasitica*, that is responsible for the devastating chestnut blight that has all but eliminated the American chestnut from North America and led to novel transgenic strategies to save this tree (Newhouse et al., 2014). In recent years, *Austropuccinia psidii* has been introduced into various regions of the world and threaten some Myrtaceae with extinction (Roux et al., 2013; Granados et al., 2017; McTaggart et al., 2017). Additionally, various pine plantations have suffered losses from pitch canker disease caused by *Fusarium circinatum* (Gordon and Reynolds, 2017). Invasive pests can also cause major loss to forestry operations. For example, introduced *Eucalyptus* plantations have experienced devastation by the gall wasp, *Leptocybe invasa* (Dittrich-Schröder et al., 2012). At the same time, global climate change will exacerbate disease and pest incidence (Sturrock et al., 2011; Das et al., 2016), as is the case with the devastating outbreaks of the mountain pine beetle (Six et al., 2018). The rapid increase in both biotic stress factors in many forests and forest plantations require a matching increase in options to mitigate their impact.

Plant defenses are complex and multiple signaling pathways may be induced following recognition of the attacker through effectors or molecular patterns specific to the invader (Jones and Dangl, 2006; reviewed in Naidoo et al., 2014). Resistance genes interact with effectors to induce effector-triggered immunity (ETI), accompanied by a hypersensitive response and containment of the invader. In the absence of such specific pathogen recognition, a basal level of defense may be induced during pathogenesis, although this may be too weak to fight off the invading microbe. Plant biotic stress interactions have been dissected in model herbaceous plants and have extended our knowledge of the sophisticated defense systems to different invaders. A challenge in forest trees is that the underlying resistance mechanisms are not always the same as described or predicted in model systems necessitating study of defense pathways and strategies utilized by tree species directly. Resistance may be polygenic (quantitative) or the result of a single major gene (qualitative; reviewed in Kovalchuk et al., 2013). The latter type of resistance may become ineffective over time due to rapidly evolving pathogens. Durable resistance that persists over time and across environments is needed in long-lived forest trees.

Long-term solutions to biotic stress interactions are clearly needed, and an important component is the inclusion of strategies that utilize plant-encoded genetic resistance to a particular stress. Genomic resources for forest trees have supported tree breeding and selection efforts in the form of genome-wide association studies (GWAS) and genomic selection (GS). In addition, genomic resources have improved our ability to infer defense pathways and genes important for producing superior forest trees with resilience against biotic stress. In this minireview, we will highlight recent discoveries in tree systems employing such approaches and propose a way forward for the insightful selection of candidate genes for genetic engineering and improvement of trees.

### Breeding for Resistance

**Figure 1:**
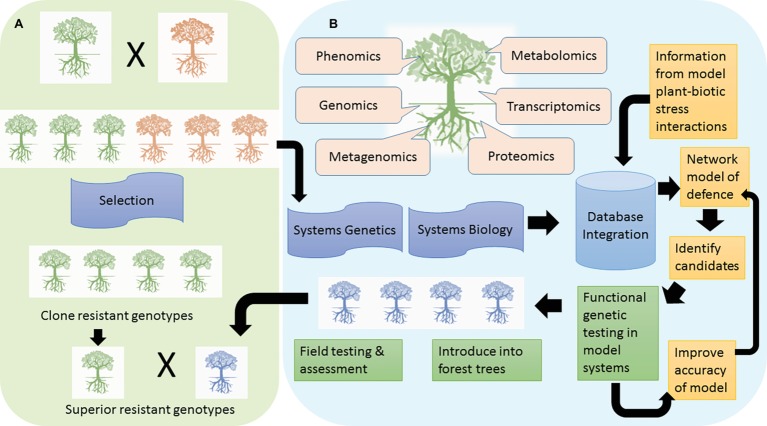
Two avenues to achieving resistance in forest trees are depicted. In tree breeding approaches, **(A)** resistant phenotypes are identified in field and tested under controlled conditions. Such resistant genotypes (green trees) are selected as parents in breeding programs to introduce resistance into a susceptible background (orange trees). Marker-assisted selection has supported plant breeding programs and new genomic tools could enhance selection of resistant trees, e.g. GWAS and GS. In the genetic engineering approach **(B)**, structured breeding programs provide the information about individual resistant or susceptible genotypes that are investigated using a multitude of omic approaches to identify defense mechanisms governing resistance and the power lies in the integration of such studies to a systems level. In addition, summation of such studies into a user-friendly database can assist with comparisons to identify genes and pathways especially important for resistance against specific challenges. A network analysis across species provides support for key genes or pathways to manipulate. The next steps are to test the function of such genes in model systems, and this information is updated in the database. Successful candidates conferring a degree of resistance can be introduced into the forest tree of interest with intense laboratory and field testing (blue trees). The transgenic tree conferring resistance may be bred into various clones selected through breeding programs to pyramid resistance.

Conventional tree breeding, based on phenotype selection, has been a successful means of attaining resistance against pests and pathogens (Figure 1A). Sniezko and Koch (2017) review some examples of successful resistance breeding programs around the world. An example from Africa is the hybridization of *Eucalyptus grandis* with *Eucalyptus urophylla* which has improved resistance against the fungal pathogen, *Chrysoporthe austroafricana* (Wingfield, 2003).

Several resistance loci have been identified in forest trees, e.g. *Ppr1* (*Puccinia psidii* resistance 1, Junghans et al., 2003) and Fusiform rust based on mapping using molecular markers. Despite this, the marker-aided selection (MAS) approach in forest systems was limited as most studies reported quantitative trait loci (QTL) discovery but were not validated for the application of MAS itself (reviewed in Sniezko and Koch, 2017) and has recently been superseded by genomic selection (GS). Grattapaglia et al. (2018) provide a critical review of GS in this special issue. In GS, associations are made between phenotypes and genotypes of a structured training population. A predictive model is made incorporating as many desirable traits as possible. The model is refined based on additional populations and used to predict desired phenotypes based on genotypic information only (Isik, 2014).

In GWAS, associations are made between the phenotype and the genotype in a structured breeding population (Isik, 2014). It is imperative that the associations are validated in a second population. The method relies on a high density of markers and a large discovery population; however, owing to limited genome information, the former is not always possible. In the case of white pine blister rust, a preliminary analysis using a small set of single-nucleotide polymorphism (SNP) markers was undertaken to identify partial resistance to the disease in *Pinus lambertiana* (Vázquez-Lobo et al., 2017). Significant associations were shown for four of the SNP markers. In Norway spruce, exon-capture sequencing was applied genome wide to identify genes associated with susceptibility to *Heterobasidion parviporum* (Mukrimin et al., 2018). Muchero et al. (2018) found *Populus trichocarpa* loci associated with the response to the fungus, *Sphaerulina musiva,* based on genome resequencing. Three loci were associated with resistance and one, encoding a G-type D-mannose–binding receptor-like kinase, was associated with susceptibility. This example demonstrates that candidate defense or susceptibility genes can be identified in a GWAS. In the case of SNP markers, candidate genes within the genomic loci associated with resistance may be inferred based on genome sequence and further information regarding the relevance of the gene during a biotic stress challenge can be determined using functional genomics, e.g. transcriptomics or proteomics. If genes in these associated regions are differentially expressed under the biotic stress challenge, it is possible that they may be contributing to the resistance phenotype.

The approaches can be even more sophisticated using regional heritability mapping (RHM). RHM uncovers variance not accounted for in GWAS as the approach examines short segments of the genome combining the effects of rare and common SNP variants between individuals to estimate the trait variance explained by such regions (Nagamine et al., 2012). Resende et al. (2017) compared RHM and GWAS approaches in *Eucalyptus* for trait mapping of wood and *Puccinia psidii* disease phenotypes. RHM was considered superior revealing more genomic regions that could be interrogated for underlying candidate defense genes. Additionally, the authors suggest that the genomic architecture revealed by the RHM-QTLs could be used to enhance genomic selection models.

An exciting prospect in forest trees would be to follow the approach of Thoen et al. (2017) who performed a GWAS in *Arabidopsis* to 11 different stresses separately and examined combinations of biotic and abiotic stress responses as a phenotype to explore the genetic architecture that underlies plant immunity. Contrasting and overlapping SNP associations were identified in biotic and abiotic stress combinations and it would be interesting to see if similar patterns are apparent in forest trees for which high-density SNP markers are available, e.g. *Eucalyptus*.

### Genetic Engineering Tree Resistance

In the absence of natural genetic variation for resistance against a particular biotic stress, genetic engineering may become necessary. The steps toward engineering resistance include gene discovery, candidate gene selection, transformation, and testing (Figure 1B). Gene discovery has been aided by omics approaches in forest trees.

#### Comparative Genomics

A trend observed in the recent genomes of tree species suggests an increase in the number of NLR genes in comparison to herbaceous plants. This may be attributed to their increased exposure to more pathogens during their longer lifetime (Yang et al., 2008; Neale et al., 2017). Specific pathogenesis-related genes were also expanded in different forest tree species such as *Pinus tecnumanii* compared to other sequenced genomes (Visser et al., 2018).

#### Transcriptomics and Proteomics

Host-pathogen interaction studies have benefitted from the use of transcriptomic approaches and have gained popularity in tree species where comparisons are made between species with disparate tolerance to pathogens (Mangwanda et al., 2015; Meyer et al., 2016). The dual-RNA sequencing approach allows for the intimate study of pathogen and pest as transcripts in both organisms can be captured simultaneously (Naidoo et al., 2017). Host tree responses including defense mechanisms and the corresponding virulence mechanisms in the pathogen can be identified. Some examples are studies on *Notholithocarpus densiflorus* and the oomycete, *Phytophthora ramorum* (Hayden et al., 2014), *Populus* and the fungal pathogen, *Septoria musiva* (Liang et al., 2014), *E. grandis* and *Chrysoporthe austroafricana* as well as *Eucalyptus nitens* and *Phytophthora cinnamomi* (Mangwanda et al., 2015; Meyer et al., 2016). Proteomics offer another level of functional relevance to genes involved in plant defense and iTRAQ (Isobaric Tags for Relative and Absolute Quantitation) was applied in *Eucalyptus* challenged with *C. austroafricana* and *Calonectria pseudoreteaudii* (Chen et al., 2015; Zwart et al., 2017). In the case of the interaction with *C. austroafricana*, proteins specific to cell death, salicylic acid signaling and systemic resistance were more apparent compared to the transcriptomic study (Mangwanda et al., 2015). Thus, proteomics may provide novel insight into plant-microbe interactions as there is not a one-to-one relationship between transcript levels and protein abundance.

#### Metagenomics

Metagenomic studies in forest trees have typically found thousands of taxa associated with individual trees from tropical forests to the boreal region and have linked the importance of these microbiomes to tree health (e.g. Kemler et al., 2013; Kembel and Mueller, 2014; Hacquard and Schadt, 2015; Agler et al., 2016; Jakuschkin et al., 2016; Vivas et al., 2017; Bullington et al., 2018). Above- and below-ground endophytic microbes can contribute to host immunity through multiple direct and indirect (*via* the host) interactions. Indirectly, endophytes can impact pathogens and pests through changes in the host physiology that affect growth or priming of the immune response *via* induced systemic resistance (ISR) and plant volatile production (Hardoim et al., 2015; Brader et al., 2017). Endophytic microbes can also directly impact pathogens and pests directly through competition, exclusion, and antibiosis (Agler et al., 2016; Bamisile et al., 2018). A further mechanism by which endophytic microbes, especially mycorrhizal fungi, may affect plant pathogens and pests is through altered nutritional status of the plant (Gange et al., 2005; Lehr et al., 2007; Pfabel et al., 2012; Kaling et al., 2018). In future, therefore, we need to improve the depth of our understanding on how mutualistic colonization of tree tissues impact pest and pathogen interactions by combining a range of omic approaches to understand the molecular mechanisms at play.

Advances in our knowledge of mechanisms by which beneficial microbes play a role in plant health as well as in our understanding of holistic plant systems are opening up new avenues whereby we can begin to consider actively selecting tree genotypes that specifically foster microbiomes to improve plant health (Mueller and Sachs, 2015). Past research has demonstrated that it is possible to control various aspects of the root and/or foliar microbiome through genetic manipulation of different metabolic pathways within the tree through single-gene alterations (Beckers et al., 2016; Veach et al., 2018). While these examples are a proof of concept that minute genomic changes to a tree’s genome can have significant impacts on microbial populations, the question now remains as to whether we can intentionally breed or engineer tree genotypes that specifically target and foster/repress the growth of specific microbes within plant tissues. While much research is needed within this area for tree species of economic importance, advances in this area have been made in agricultural crops (Besserer et al., 2006; Fierer et al., 2007; Carvalhais et al., 2015). Should similar pathways be targeted in tree systems we might be able to similarly improve tree health through naturally present microbial populations.

#### Metabolomics

Metabolomics in forest tree-biotic stress interactions have been limited to the study of specific secondary metabolites, e.g. terpenes (Oates et al., 2015; Naidoo et al., 2018), using methods such gas or liquid chromatography coupled to mass spectrometry (GC-MS and LC-MS, respectively) and near-infrared reflectance (NIR) technologies. Metabolomics in European common ash, *Fraxinus excelsior*, revealed different profiles associated with resistance and tolerance to the fungus, *Hymenoscyphus fraxineus* (Sambles et al., 2017). Where possible, combining different approaches, e.g. transcriptomics and metabolomics, enhances the selection of pathways implicated in defense. This was applied in pedunculate oak (Kersten et al., 2013). Oaks resistant and susceptible to the leaf roll miner herbivore had specific volatile profiles and susceptible trees produced a volatile blend more attractive for the pest. At the transcriptome level, resistance was attributed to constitutive rather than induced defenses. Plant-plant interactions play an important role in defense signaling against pathogens (for a recent review, please see Subrahmaniam et al., 2018 who describe candidate genes defining plant-plant interactions as cell wall modifications and defense pathways). This phenomenon has been studied in trees to a limited extent as indirect defenses. Indirect defenses involve the release of volatile compounds that attract natural enemies of the pest or signal neighboring plants of the threat (Unsicker et al., 2009). This has been demonstrated in elm (*Ulmus minor*) responses to the elm leaf beetle, *Xanthogaleruca luteola,* where oviposition altered volatile profiles that attracted the specialist egg parasitoid, *Oomyzus gallerucae* (Meiners and Hilker, 2000). *Eucalyptus* also uses these signals to prime neighboring plants, e.g. feeding by *Ctenarytaina eucalypti* induced volatile responses which increased defense-related compounds in unwounded plants (Troncoso et al., 2012). Oviposition or herbivory-induced chemical cues released by plants have been shown to serve as reliable indicators of the presence of prey to predators and parasitoids of the pest (Hilker et al., 2002; Bruinsma et al., 2009).

While these previous applications have proceeded at a rapid rate in forestry research, an area that still lags behind is phenomics. Phenomics involves the high-throughput capture of the phenotypes of an organism, influenced by its genotype and genotype × environment. Phenomics informs the genotype-phenotype map (reviewed in Houle et al., 2010) and new imaging technologies are being pursued to achieve this goal. Ludovisi et al. (2017) describe the use of unmanned aerial vehicle type of remote sensing and imaging to enable high-throughput field phenotyping. The authors applied this to black poplar populations to capture precise phenotypes for drought responses. These techniques are equally promising for biotic stress phenotyping to accelerate the development of superior genotypes.

With the generation of multiple levels of omics data, systems biology views of organisms become possible. This provides a framework for linking molecular interactions with complex traits (Mizrachi et al., 2017). The integration of information from different experiments can be used to generate a network modeling the dynamics and complexity of a biological system. The development of such models is iterative and is used to refine new models. Studies in *Arabidopsis* show the power of this approach to uncover key mechanisms important in plant defense (Mukhtar et al., 2011). A related approach is systems genetics (also known as genetical genomics) which incorporates the genetic variation of organisms with the systems level phenotyping to dissect complex traits (Jansen and Nap, 2001). Tree breeding programs provide the structured populations for such types of studies (Figure 1B). Molecular mechanisms underlying wood properties in *Eucalyptus* were characterized in this manner (Mizrachi et al., 2017), with systems genetics studies involving tree biotic stress interactions not far behind.

## Selection of Candidate Defense Genes

The wealth of omics data that is being generated for trees during pest and pathogen challenge requires concomitant development of resources to promote access by the scientific community. User-friendly databases are increasingly becoming available to analyze and visualize omics data in woody perennials, which can assist with identifying key genes and pathways for resistance. The TreeGenes database (Wegrzyn et al., 2008^1^) is a forest tree genomics resource for integration and analysis of genome sequences, transcriptomes, genetic maps, molecular markers, and phenotypic data for 1,749 species. The Plant Genome Integrative Explorer (Sundell et al., 2015^2^) provides access to genomic and transcriptomic data for *Poplar*, *Arabidopsis*, *Eucalyptus*, and conifer. PlantGenIE contains tools for expression analyses and visualization of gene co-expression networks within and across forest species (Netotea et al., 2014). Gramene (Tello-Ruiz et al., 2016^3^) contains the plant reactome database for analysis of plant metabolic and regulatory pathways. MorphDB (Zwaenepoel et al., 2018^4^) assists in identifying missing genes in pathways and regulators such as transcription factors or other signaling genes. Such approaches can place the potential resistance genes in a functional context relative to known resistance genes and thus assist in identifying and prioritizing candidates (Rhee and Mutwil, 2014). Flowing out of such databases should be the development of a predictive network model to understand the potential functional role of genes within a pathway (Figure 1B). Mewalal et al. (2014) provides an insightful review on identifying and prioritizing genes using omic approaches to assist new hypothesis-driven experiments. Once candidate genes are prioritized and selected, they are tested in model systems for a disease or pest tolerant phenotype. This has been demonstrated for *P. trichocarpa* whereby overexpression of a salicylic acid-inducible gene, *PtrWRKY73,* in *Arabidopsis* increased resistance to *Pseudomonas syringae* (biotroph) but reduced resistance to *Botrytis cinerea* (necrotroph) (Duan et al., 2015). Another study made use of *Nicotiana tabacum* overexpressing antimicrobial protein Sp-AMP2 (PR-19) from *Pinus sylvestris* L., which enhanced resistance to *B. cinerea* (Jaber et al., 2017). Following successful or unsuccessful demonstration of a candidate gene’s function, the model can be refined followed by repeated testing until a desired phenotype is observed (Figure 1B). Then introduction of the gene into the desired forest tree species is pursued *via* genetic transformation. Candidate genes may be overexpressed, knocked down, or knocked out. Jiang et al. (2017) utilized CRISPR/Cas9 knockouts of candidate *WRKY* transcription factors to unravel regulatory mechanisms in *Populus* during interaction with *Melampsora* rust. Once successful transgenic lines are obtained, extensive functional testing under different conditions is made to determine if the candidate gene confers biotic stress resistance. One limitation of producing transgenic trees is that transformation may be optimized for specific clonal backgrounds. This can be circumvented by incorporating transgenic trees as parents in breeding programs to transfer the desired resistant phenotype to other genetic backgrounds (Figure 1).

## Advances Toward Engineering Resistance

Forest biotechnology companies have been developing transgenic forest tree species, *Populus* and *Eucalyptus*, to enhance biomass and resistance to stresses. ArborGen Inc. has developed transgenic freeze-tolerant *Eucalyptus* (Zhang et al., 2012) and SweTree Technologies have developed transgenic hybrid aspen (*Populus tremula* × *Populus tremuloides*) with enhanced growth properties (Eriksson et al., 2006). FuturaGene Ltd. has developed and commercialized the first genetically modified *Eucalyptus* tree with 20% more biomass (“Brazil approves transgenic eucalyptus,” 2015) with developments to commercialize disease-resistant trees (Avisar et al., 2013) underway. Additionally, genome editing holds great promise for accelerated breeding in forestry (Bewg et al., 2018). Therefore, once we identify genes or metabolic pathways crucial to disease resistance, we will be able to quickly manipulate these pathways and produce plants that are available to industry in a timelier manner than ever before.

## Conclusion

With the unprecedented increase in forest pests and pathogens, the two avenues to generate resistant trees are necessary. Breeding and genetic engineering could be combined to accelerate achieving the goal of resistance in forestry. Genomic tools are enhancing our rate of gene discovery in forest trees; however, the vast amounts of data must be more centralized and accessible to identify candidate genes for testing. As is the trend in model systems, tree immunity should also be increasingly viewed from a holistic, systems-based perspective that incorporates the complexity of the biotic interactions outside, within and below the tree. This includes the potential for direct manipulation of host-associated microbes for tree resistance, as well as the recognition that genetic engineering of the host impacts the microbiome with potentially important consequences for tree resistance. A concerted effort has to be made to make engineering resistance a reality in forestry.

## Author Contributions

SN determined the scope of the review. SN, BS, JMP, CNO and DC wrote different sections of the review and contributed equally. SN and CNO designed the figure and all authors contributed to final edits.

### Conflict of Interest Statement

The authors declare that the research was conducted in the absence of any commercial or financial relationships that could be construed as a potential conflict of interest.

## Footnotes

^1^https://treegenesdb.org/Drupal/

^2^http://plantgenie.org/

^3^http://www.gramene.org/

^4^http://bioinformatics.psb.ugent.be/webtools/morphdb/morphDB/index/

## References

[ref1] Agler M. T. Ruhe J. Kroll S. Morhenn C. Kim S. T. Weigel D. (2016). Microbial hub taxa link host and abiotic factors to plant microbiome variation. PLoS Biol. 14, 1–31. 10.1371/journal.pbio.1002352 PMC4720289 26788878

[ref2] Avisar D. Stein H. Shani Z. (2013). Pest-resistant plants containing a combination of a spider toxin and a chitinase. Google Patents.

[ref3] Bamisile B. S. Dash C. K. Akutse K. S. Keppanan R. Wang L. (2018). Fungal endophytes: beyond herbivore management. Front. Microbiol. 9, 1–11. 10.3389/fmicb.2018.00544 29628919 PMC5876286

[ref4] Beckers B. Op De Beeck M. Weyens N. Van Acker R. Van Montagu M. Boerjan W.. (2016). Lignin engineering in field-grown poplar trees affects the endosphere bacterial microbiome. Proc. Natl. Acad. Sci. 113, 2312–2317. 10.1073/pnas.1523264113, PMID: 26755604 PMC4776533

[ref5] Besserer A. Puech-Pagès V. Kiefer P. Gomez-Roldan V. Jauneau A. Roy S. (2006). Strigolactones stimulate arbuscular mycorrhizal fungi by activating mitochondria. PLoS Biol. 4, 1239–1247. 10.1371/journal.pbio.0040226 PMC1481526 16787107

[ref6] Bewg W. P. Ci D. Tsai C.-J. (2018). Genome editing in trees: from multiple repair pathways to long-term stability. Front. Plant Sci. 9:1732. 10.3389/fpls.2018.01732 30532764 PMC6265510

[ref7] Brader G. Compant S. Vescio K. Mitter B. Trognitz F. Ma L.-J.. (2017). Ecology and genomic insights into plant-pathogenic and plant-nonpathogenic endophytes. Annu. Rev. Phytopathol. 55, 61–83. 10.1146/annurev-phyto-080516-035641, PMID: 28489497

[ref8] Bruinsma M. Posthumus M. A. Mumm R. Mueller M. J. Van Loon J. J. A. Dicke M. (2009). Jasmonic acid-induced volatiles of *Brassica oleracea* attract parasitoids: Effects of time and dose, and comparison with induction by herbivores. J. Exp. Bot. 60, 2575–2587. 10.1093/jxb/erp101, PMID: 19451186 PMC2692006

[ref9] Bullington L. S. Lekberg Y. Sniezko R. Larkin B. (2018). The influence of genetics, defensive chemistry, and the fungal microbiome on disease outcome in whitebark pine trees. Mol. Plant Pathol. 19, 1847–1858. 10.1111/mpp.12663 PMC6638087 29388309

[ref10] Carvalhais L. C. Dennis P. G. Badri D. V. Kidd B. N. Vivanco J. M. Schenk P. M. (2015). Linking jasmonic acid signalling, root exudates and rhizosphere microbiomes. Mol. Plant-Microbe Interact. 28, 1049–1058. 10.1094/MPMI-01-15-0016-R, PMID: 26035128

[ref11] Chen Q. Guo W. Feng L. Ye X. Xie W. Huang X.. (2015). Data for transcriptome and proteome analysis of Eucalyptus infected with *Calonectria pseudoreteaudii*. Data Br. 3, 24–28. 10.1016/j.dib.2014.12.008, PMID: 26217712 PMC4509981

[ref12] Das T. Majumdar M. H. D. Devi R. K. T. Rajesh T. (2016). Climate change impact on plant diseases. SAARC J. Agric. 14, 200–209. 10.3329/sja.v14i2.31259, PMID: 28638211

[ref13] Dittrich-Schröder G. Wingfield M. J. Hurley B. P. Slippers B. (2012). Diversity in *Eucalyptus* susceptibility to the gall-forming wasp *Leptocybe invasa*. Agric. For. Entomol. 14, 419–427. 10.1111/j.1461-9563.2012.00583.x

[ref14] Duan Y. Jiang Y. Ye S. Karim A. Ling Z. He Y.. (2015). PtrWRKY73, a salicylic acid-inducible poplar WRKY transcription factor, is involved in disease resistance in *Arabidopsis thaliana*. Plant Cell Rep. 34, 831–841. 10.1007/s00299-015-1745-5, PMID: 25627252 PMC4405351

[ref16] Eriksson M. Moritz T. Israelsson M. Olsson O. (2006). Transgenic trees exhibiting increased growth, biomass production and xylem fibre length, and methods for their production. Google Patents. 10.1038/77355 10888850

[ref17] Fierer N. Bradford M. A. Jackson R. B. (2007). Toward an ecological classification of soil bacteria. Ecology 88, 1354–1364. 10.1007/s00209-018-2115-0, PMID: 17601128

[ref18] Gange A. C. Gane D. R. J. Chen Y. Gong M. (2005). Dual colonization of *Eucalyptus urophylla* S.T. Blake by arbuscular and ectomycorrhizal fungi affects levels of insect herbivore attack. Agric. For. Entomol. 7, 253–263. 10.1111/j.1461-9555.2005.00268.x

[ref19] Gordon T. R. Reynolds G. J. (2017). Plasticity in plant-microbe interactions: a perspective based on the pitch canker pathosystem. Phytoparasitica 45, 1–8. 10.1007/s12600-016-0558-6

[ref20] Granados G. M. McTaggart A. R. Barnes I. Rodas C. A. Roux J. Wingfield M. J. (2017). The pandemic biotype of *Austropuccinia psidii* discovered in South America. Australas. Plant Pathol. 46, 267–275. 10.1007/s13313-017-0488-x

[ref21] Grattapaglia D. Silva-Junior O. B. Resende R. T. Cappa E. P. Müller B. S. F. Tan B.. (2018). Quantitative Genetics and Genomics Converge to Accelerate Forest Tree Breeding. Front. Plant Sci. 9. 10.3389/fpls.2018.01693, PMID: 30524463 PMC6262028

[ref22] Hacquard S. Schadt C. W. (2015). Towards a holistic understanding of the beneficial interactions across the Populus microbiome. New Phytol. 205, 1424–1430. 10.1111/nph.13133, PMID: 25422041

[ref23] Hardoim P. R. van Overbeek L. S. Berg G. Pirttilä A. M. Compant S. Campisano A.. (2015). The hidden world within plants: ecological and evolutionary considerations for defining functioning of microbial endophytes. Microbiol. Mol. Biol. Rev. 79, 293–320. 10.1128/MMBR.00050-14, PMID: 26136581 PMC4488371

[ref24] Hayden K. J. Garbelotto M. Knaus B. J. Cronn R. C. Rai H. Wright J. W. (2014). Dual RNA-seq of the plant pathogen *Phytophthora ramorum* and its tanoak host. Tree Genet. Genomes 10, 489–502. 10.1007/s11295-014-0698-0

[ref25] Hilker M. Kobs C. Varama M. Schrank K. (2002). Insect egg deposition induces *Pinus sylvestris* to attract egg parasitoids. J. Exp. Biol. 205, 455–461. 10.1242/jeb.00732, PMID: 11893759

[ref26] Houle D. Govindaraju D. R. Omholt S. (2010). Phenomics: the next challenge. Nat. Rev. Genet. 11, 855. 10.1038/nrg2897, PMID: 21085204

[ref27] Hurley B. P. Garnas J. Wingfield M. J. Branco M. Richardson D. M. Slippers B. (2016). Increasing numbers and intercontinental spread of invasive insects on eucalypts. Biol. Invasions 18, 921–933. 10.1007/s10530-016-1081-x

[ref28] Isik F. (2014). Genomic selection in forest tree breeding: the concept and an outlook to the future. New For. 45, 379–401. 10.1007/s11056-014-9422-z

[ref29] Jaber E. Kovalchuk A. Raffaello T. Keriö S. Teeri T. Asiegbu F. O. (2017). A gene encoding scots pine antimicrobial protein Sp-AMP2 (PR-19) confers increased tolerance against *Botrytis cinerea* in transgenic tobacco. Forests 9, 1–15. 10.3390/f9010010

[ref30] Jakuschkin B. Fievet V. Schwaller L. Fort T. Robin C. Vacher C. (2016). Deciphering the pathobiome: intra- and interkingdom interactions involving the pathogen *Erysiphe alphitoides*. Microb. Ecol. 72, 870–880. 10.1007/s00248-016-0777-x, PMID: 27147439

[ref31] Jansen R. C. Nap J. P. (2001). Genetical genomics: the added value from segregation. Trends Genet. 17, 388–391. 10.1016/S0168-9525(01)02310-1, PMID: 11418218

[ref32] Jiang Y. Guo L. Ma X. Zhao X. Jiao B. Li C.. (2017). The WRKY transcription factors PtrWRKY18 and PtrWRKY35 promote *Melampsora* resistance in *Populus*. Tree Physiol. 37, 665–675. 10.1093/treephys/tpx008, PMID: 28338710

[ref33] Jones J. D. G. Dangl J. L. (2006). The plant immune system. Nature 444, 323–329. 10.1038/nature05286, PMID: 17108957

[ref34] Junghans D. T. Alfenas A. C. Brommonschenkel S. H. Oda S. Mello E. J. Grattapaglia D. (2003). Resistance to rust (*Puccinia psidii* Winter) in *Eucalyptus*: mode of inheritance and mapping of a major gene with RAPD markers. Theor. Appl. Genet. 108, 175–180. 10.1007/s00122-003-1415-9, PMID: 14504745

[ref35] Kaling M. Schmidt A. Moritz F. Rosenkranz M. Witting M. Kasper K. (2018). Mycorrhiza-triggered transcriptomic and metabolomic networks impinge on herbivore fitness. Plant Physiol. 176, 2639–2656. 10.1104/pp.17.01810 29439210 PMC5884605

[ref36] Kembel S. W. Mueller R. C. (2014). Plant traits and taxonomy drive host associations in tropical phyllosphere fungal communities. Botany 92, 303–311. 10.1139/cjb-2013-0194

[ref37] Kemler M. Garnas J. Wingfield M. J. Gryzenhout M. Pillay K. A. Slippers B. (2013). Ion torrent PGM as tool for fungal community analysis: a case study of endophytes in *Eucalyptus grandis* reveals high taxonomic diversity. PLoS One 7:e81718. 10.1371/journal.pone.0081718, PMID: 24358124 PMC3864840

[ref38] Kersten B. Ghirardo A. Schnitzler J. P. Kanawati B. Schmitt-Kopplin P. Fladung M. (2013). Integrated transcriptomics and metabolomics decipher differences in the resistance of pedunculate oak to the herbivore *Tortrix viridana* L. BMC Genomics 14:737. 10.1186/1471-2164-14-737 24160444 PMC4007517

[ref39] Kovalchuk A. Kerio S. Oghenekaro A. O. Jaber E. Raffaello T. Asiegbu F. O. (2013). Antimicrobial defences and resistance in forest trees: challenges and perspectives in a genomics era. Annu. Rev. Phytopathol. 51, 221–244. 10.1146/annurev-phyto-082712-102307, PMID: 23682916

[ref40] Lehr N. Schrey S. D. Tarkka M. T. (2007). Root inoculation with a forest soil streptomycete leads to locally and systemically increased resistance against phytopathogens in Norway spruce. New Phytol. 177, 965–976. 10.1111/j.1469-8137.2007.02322.x 18086220

[ref41] Liang H. Staton M. Xu Y. Xu T. Leboldus J. (2014). Comparative expression analysis of resistant and susceptible *Populus* clones inoculated with *Septoria musiva*. Plant Sci. 223, 69–78. 10.1016/j.plantsci.2014.03.004, PMID: 24767117

[ref42] Ludovisi R. Tauro F. Salvati R. Khoury S. Mugnozza Scarascia G. Harfouche A. (2017). UAV-based thermal imaging for high-throughput field phenotyping of black poplar response to drought. Front. Plant Sci. 8, 1–18. 10.1109/AMS.2009.139 29021803 PMC5623950

[ref43] Mangwanda R. Myburg A. A. Naidoo S. (2015). Transcriptome and hormone profiling reveals *Eucalyptus grandis* defence responses against *Chrysoporthe austroafricana*. BMC Genomics 16, 1–13. 10.1186/s12864-015-1529-x 25903559 PMC4405875

[ref44] McTaggart A. R. Shuey L. S. Granados G. M. du Plessis E. Fraser S. Barnes I.. (2017). Evidence that *Austropuccinia psidii* may complete its sexual life cycle on Myrtaceae. Plant Pathol. 67, 729–734. 10.1111/ppa.12763, PMID: 28492918

[ref45] Meiners T. Hilker M. (2000). Induction of plant synomones by oviposition of a phytophagus insect. J. Chem. Ecol. 26, 221–232. 10.1023/A:1005453830961

[ref46] Mewalal R. Mizrachi E. Mansfield S. D. Myburg A. A. (2014). Cell wall-related proteins of unknown function: missing links in plant cell wall development. Plant Cell Physiol. 55, 1031–1043. 10.1093/pcp/pcu050, PMID: 24683037

[ref47] Meyer F. E. Shuey L. S. Naidoo S. Mamni T. Berger D. K. Myburg A. A. (2016). Dual RNA-sequencing of *Eucalyptus nitens* during *Phytophthora cinnamomi* challenge reveals pathogen and host factors influencing compatibility. Front. Plant Sci. 7, 1–15. 10.3389/fpls.2016.00191 26973660 PMC4773608

[ref48] Mizrachi E. Verbeke L. Christie N. Fierro A. C. Mansfield S. D. Davis M. F.. (2017). Network-based integration of systems genetics data reveals pathways associated with lignocellulosic biomass accumulation and processing. Proc. Natl. Acad. Sci. 114, 1195–1200. 10.1073/pnas.1620119114, PMID: 28096391 PMC5293113

[ref49] Muchero W. Sondreli K. L. Chen J.-G. Urbanowicz B. R. Zhang J. Singan V. (2018). Association mapping, transcriptomics, and transient expression identify candidate genes mediating plant–pathogen interactions in a tree. Proc. Natl. Acad. Sci. 115, 11573–11578. 10.1073/pnas.1804428115 30337484 PMC6233113

[ref50] Mueller U. G. Sachs J. L. (2015). Engineering microbiomes to improve plant and animal health. Trends Microbiol. 23, 606–617. 10.1016/j.tim.2015.07.009 26422463

[ref51] Mukhtar M. S. Carvunis A.-R. Dreze M. Epple P. Steinbrenner J. Moore J.. (2011). Independently evolved virulence effectors converge onto hubs in a plant immune system network. Science 333, 596–601. 10.1126/science.1203659, PMID: 21798943 PMC3170753

[ref52] Mukrimin M. Kovalchuk A. Neves L. G. Jaber E. H. A. Haapanen M. Kirst M. (2018). Genome-wide exon-capture approach identifies genetic variants of norway spruce genes associated with susceptibility to *heterobasidion parviporum* infection. Front. Plant Sci. 9, 1–13. 10.3389/fpls.2018.00793 29946332 PMC6005875

[ref53] Nagamine Y. Pong-Wong R. Navarro P. Vitart V. Hayward C. Rudan I. (2012). Localising loci underlying complex trait variation using regional genomic relationship mapping. PLoS One 7:e46501. 10.1371/journal.pone.0046501 23077511 PMC3471913

[ref54] Naidoo S. Christie N. Acosta J. J. Mphalele M. M. Payn K. G. Myburg A. A. (2018). Terpenes associated with resistance against the gall wasp, Leptocybe invasa, in *Eucalyptus grandis*. Plant Cell Environ. 41, 1840–1851. 10.1111/pce.13323 29710389

[ref55] Naidoo S. Külheim C. Zwart L. Mangwanda R. Oates C. N. Visser E. A.. (2014). Uncovering the defence responses of eucalyptus to pests and pathogens in the genomics age. Tree Physiol. 34, 931–943. 10.1093/treephys/tpu075, PMID: 25261123

[ref56] Naidoo S. Visser E. A. Zwart L. Toit Y. d. Bhadauria V. Shuey L. S. (2017). Dual RNA-sequencing to elucidate the plant-pathogen duel. Curr. Issues Mol. Biol. 27, 127–141. 10.21775/cimb.027.127 28885179

[ref57] Neale D. B. Martínez-García P. J. De La Torre A. R. Montanari S. Wei X.-X. (2017). Novel insights into tree biology and genome evolution as revealed through genomics. Annu. Rev. Plant Biol. 68, 457–483. 10.1146/annurev-arplant-042916-041049, PMID: 28226237

[ref58] Netotea S. Sundell D. Street N. R. Hvidsten T. R. (2014). ComPlEx: conservation and divergence of co-expression networks in *A. thaliana, Populus* and *O. sativa*. BMC Genomics 15, 1–17. 10.1186/1471-2164-15-106 24498971 PMC3925997

[ref59] Newhouse A. E. Polin-McGuigan L. D. Baier K. A. Valletta K. E. R. Rottmann W. H. Tschaplinski T. J.. (2014). Transgenic american chestnuts show enhanced blight resistance and transmit the trait to T1 progeny. Plant Sci. 228, 88–97. 10.1016/j.plantsci.2014.04.004, PMID: 25438789

[ref60] Oates C. N. Külheim C. Myburg A. A. Slippers B. Naidoo S. (2015). The transcriptome and terpene profile of *Eucalyptus grandis* reveals mechanisms of defense against the insect pest, *Leptocybe invasa*. Plant Cell Physiol. 56, 1418–1428. 10.1093/pcp/pcv064, PMID: 25948810

[ref61] Pfabel C. Eckhardt K. U. Baum C. Struck C. Frey P. Weih M. (2012). Impact of ectomycorrhizal colonization and rust infection on the secondary metabolism of poplar (*Populus trichocarpa × deltoides*). Tree Physiol. 32, 1357–1364. 10.1093/treephys/tps093, PMID: 23065191

[ref62] Resende R. T. Resende M. D. V. Silva F. F. Azevedo C. F. Takahashi E. K. Silva-Junior O. B.. (2017). Regional heritability mapping and genome-wide association identify loci for complex growth, wood and disease resistance traits in *Eucalyptus*. New Phytol. 213, 1287–1300. 10.1111/nph.14266, PMID: 28079935

[ref63] Rhee S. Y. Mutwil M. (2014). Towards revealing the functions of all genes in plants. Trends Plant Sci. 19, 212–221. 10.1016/j.tplants.2013.10.006, PMID: 24231067

[ref64] Roux J. Greyling I. Coutinho T. A. Verleur M. Wingfield M. J. (2013). The Myrtle rust pathogen, *Puccinia psidii*, discovered in Africa. IMA Fungus 4, 155–159. 10.5598/imafungus.2013.04.01.14, PMID: 23898420 PMC3719202

[ref65] Sambles C. M. Salmon D. L. Florance H. Howard T. P. Smirnoff N. Nielsen L. R. (2017). Data descriptor: ash leaf metabolomes reveal differences between trees tolerant and susceptible to ash dieback disease. Sci. Data 4, 1–13. 10.1038/sdata.2017.190 PMC5735976 29257137

[ref66] Santini A. Ghelardini L. De Pace C. Desprez-Loustau M. L. Capretti P. Chandelier A.. (2013). Biogeographical patterns and determinants of invasion by forest pathogens in Europe. New Phytol. 197, 238–250. 10.1111/j.1469-8137.2012.04364.x, PMID: 23057437

[ref67] Six D. L. Vergobbi C. Cutter M. (2018). Are survivors different? Genetic-based selection of trees by mountain pine beetle during a climate change-driven outbreak in a high-elevation pine forest. Front. Plant Sci. 9, 1–11. 10.3389/fpls.2018.00993 30083173 PMC6064936

[ref68] Sniezko R. A. Koch J. (2017). Breeding trees resistant to insects and diseases: putting theory into application. Biol. Invasions 19, 3377–3400. 10.1007/s10530-017-1482-5

[ref69] Sturrock R. N. Frankel S. J. Brown A. V. Hennon P. E. Kliejunas J. T. Lewis K. J. (2011). Climate change and forest diseases. Plant Pathol. 60, 133–149. 10.1111/j.1365-3059.2010.02406.x

[ref70] Subrahmaniam H. J. Libourel C. Journet E. P. Morel J. B. Muños S. Niebel A.. (2018). The genetics underlying natural variation of plant–plant interactions, a beloved but forgotten member of the family of biotic interactions. Plant J. 93, 747–770. 10.1111/tpj.13799, PMID: 29232012

[ref71] Sundell D. Mannapperuma C. Netotea S. Delhomme N. Lin Y. C. Sjödin A.. (2015). The plant genome integrative explorer resource: PlantGenIE.org. New Phytol. 208, 1149–1156. 10.1111/nph.13557, PMID: 26192091

[ref72] Tello-Ruiz M. K. Stein J. Wei S. Preece J. Olson A. Naithani S.. (2016). Gramene 2016: comparative plant genomics and pathway resources. Nucleic Acids Res. 44, D1133–D1140. 10.1093/nar/gkv1179, PMID: 26553803 PMC4702844

[ref73] Thoen M. P. M. Davila Olivas N. H. Kloth K. J. Coolen S. Huang P. P. Aarts M. G. M.. (2017). Genetic architecture of plant stress resistance: multi-trait genome-wide association mapping. New Phytol. 213, 1346–1362. 10.1111/nph.14220, PMID: 27699793 PMC5248600

[ref74] Troncoso C. Becerra J. Perez C. Hernandez V. Martin A. Sanchez-olate M. (2012). Induction of defensive responses in *Eucalyptus globulus* (Labill) plants, against *Ctenarytaina eucalypti* (Maskell) (Hemiptera: Psyllidae). Am. J. Plant Sci. 2012, 589–595. 10.4236/ajps.2012.35071

[ref75] Unsicker S. B. Kunert G. Gershenzon J. (2009). Protective perfumes: the role of vegetative volatiles in plant defense against herbivores. Curr. Opin. Plant Biol. 12, 479–485. 10.1016/j.pbi.2009.04.001, PMID: 19467919

[ref76] Vázquez-Lobo A. De La Torre A. R. Martínez-García P. J. Vangestel C. Wegzryn J. L. Ćalić I. (2017). Finding loci associated to partial resistance to white pine blister rust in sugar pine (*Pinus lambertiana* Dougl.). Tree Genet. Genomes 13:108. 10.1007/s11295-017-1190-4

[ref77] Veach A. M. Yip D. Engle N. L. Yang Z. K. Bible A. Morrell-Falvey J. (2018). Modification of plant cell wall chemistry impacts metabolome and microbiome composition in *Populus PdKOR1* RNAi plants. Plant Soil 429, 349–361. 10.1007/s11104-018-3692-8

[ref78] Visser E. A. Wegrzyn J. L. Myburg A. A. Naidoo S. (2018). Defence transcriptome assembly and pathogenesis related gene family analysis in *Pinus tecunumanii* (low elevation). BMC Genomics 19, 1–13. 10.1186/s12864-018-5015-0 30139335 PMC6108113

[ref79] Vivas M. Kemler M. Mphahlele M. M. Wingfield M. J. Slippers B. (2017). Maternal effects on phenotype, resistance and the structuring of fungal communities in *Eucalyptus grandis*. Environ. Exp. Bot. 140, 120–127. 10.1016/j.envexpbot.2017.06.002

[ref80] Wegrzyn J. L. Lee J. M. Tearse B. R. Neale D. B. (2008). TreeGenes: a forest tree genome database. Int. J. Plant Genomics 2008, 1–7. 10.1155/2008/412875, PMID: 18725987 PMC2517852

[ref81] Wingfield M. J. (2003). Daniel McAlpine Memorial Lecture: increasing threat of diseases to plantation forests in the Southern Hemisphere: lessons from *Cryphonectria* canker. Australas. Plant Pathol. 32, 133–139. 10.1071/AP03024

[ref82] Wingfield M. J. Brockerhoff E. G. Wingfield B. D. Slippers B. (2015). Planted forest health: the need for a global strategy. Science 349, 832–836. 10.1126/science.aac6674, PMID: 26293956

[ref83] Yang S. Zhang X. Yue J. X. Tian D. Chen J. Q. (2008). Recent duplications dominate NBS-encoding gene expansion in two woody species. Mol. Gen. Genomics. 280, 187–198. 10.1007/s00438-008-0355-0, PMID: 18563445

[ref84] Zhang C. Kwan B. Chang S. Raymond P. (2012). Freeze tolerant eucalyptus. Google Patents.

[ref86] Zwaenepoel A. Diels T. Amar D. Van Parys T. Shamir R. Van de Peer Y. (2018). MorphDB: prioritizing genes for specialized metabolism pathways and gene ontology categories in plants. Front. Plant Sci. 9, 1–13. 10.3389/fpls.2018.00352 29616063 PMC5867296

[ref87] Zwart L. Berger D. K. Moleleki L. N. Van Der Merwe N. A. Myburg A. A. Naidoo S. (2017). Evidence for salicylic acid signalling and histological changes in the defence response of *Eucalyptus grandis* to *Chrysoporthe austroafricana*. Sci. Rep. 7, 1–12. 10.1038/srep45402 28349984 PMC5368643

